# COVID-19 and the worldwide actions to mitigate its effects using 3D printing

**DOI:** 10.2217/3dp-2021-0004

**Published:** 2021-03-24

**Authors:** Guilherme Arthur Longhitano, Jorge Vicente Lopes da Silva

**Affiliations:** 1 ^1^Laboratory for Three-Dimensional Technologies, Center for Information Technology Renato Archer (CTI), 13069 901 Campinas, Brazil; 2 ^2^School of Chemical Engineering, University of Campinas, 13083 852 Campinas, Brazil; 3 ^3^National Institute of Biofabrication (INCT-BIOFABRIS), 13083 852 Campinas, Brazil

**Keywords:** 3D printing, additive manufacturing, coronavirus, COVID-19, medical devices, pandemic, PPE

## Abstract

#3Dprinting assumed a fundamental role during the #covid-19 #pandemic worldwide. It establishes as a notorious manufacturing technique in the industry and society and a powerful ally for #future emergencies.

## The COVID-19 pandemic & 3D printing

More than a year has passed since the coronavirus disease (COVID-19) had its first patient documented on 19 December 2019 [1]. According to the COVID-19 Map from Johns Hopkins Coronavirus Resource Center, the disease spread through 192 countries, infecting more than 121 million people and killing more than 2.68 million until 18 March 2021 [2]. Countries face a second or even a third wave, new measures are being taken, and the severe acute respiratory syndrome coronavirus-2 does not seem to cease. As the vaccination programs have begun worldwide, people can finally expect a light at the end of the tunnel.

Simultaneously, the high number of patients created a high demand for personal protection equipment (PPE), medical devices and raw material during the first months of the pandemic. The social distancing and closure of diverse industrial segments created supply chain and logistics disruptions, leading to a critical lack of essential items in hospitals [3]. The COVID-19 pandemic numbers are astonishing but certainly could be higher if the 3D printing community did not help in the fight against the disease.

When this issue came up, individual makers, groups, universities and companies quickly responded, starting to provide 3D printed parts. The high availability of desktop 3D printers due to patents’ expiration and lowering in prices in the last decade [4], resulting in open source projects and files found in repository websites [4], such as Thingverse and NIH 3D Print Exchange, and also in social media [5], made 3D printing, or additive manufacturing, as an immediate alternative for the production of innovative devices and parts to fight the COVID-19 pandemic.

## 3D printed devices

The lack of PPE, medical devices and the emergence of new types of habits, such as avoiding touching surfaces and wearing face masks by the general public [6], demanded an instantaneous response for providing these items in all countries.

In one of the most notorious cases, the ISINNOVA company from Italy created a 3D printed connector valve and splitter named *Charlotte* and *Dave* [7], respectively. These parts were engineered to be fabricated by fused filament fabrication (FFF) 3D printing technique and used with full-face snorkeling masks to help patients with hypoxia symptoms undergo noninvasive ventilation without contaminating the ambient.

Another relevant case was PPE production. Face shields, a type of PPE used to protect the user from contamination from airborne particles due to splashes and spray, are classified as Class I medical devices by the US FDA regulatory body. This low-risk classification, combined with the high demand of these items, and the social media divulgation, leveraged its production in universities, companies, and makerspaces, boosting this PPE as the most produced item by the 3D printing community [4].

In the diagnosis area, nasopharyngeal test swabs for reverse transcription-PCR tests were designed. These FDA Class I medical devices were designed with complex structures, only achievable by 3D printing. They were validated to be produced by the stereolithography 3D printing technique [8], turning into some companies’ final product.

Other types of 3D printed items include Venturi valves, face masks, ear savers, reposition or adaptation parts for medical devices, isolation chambers and field respirators, among others. All these items presented significant importance in fighting the COVID-19 pandemic, and more extensive data can be found in recent reviews [9–11].

## Fighting the COVID-19 pandemic in Brazil

In our research center in Brazil, at the beginning of the COVID-19 pandemic in the country, we worked in partnership with a multidisciplinary volunteer team from a non-governmental organization formed by physicians, engineers, designers and technicians, to produce face shields and *Charlotte* valves. In both cases, we used 3D printing first as a rapid prototyping tool, achieving an optimized model, which was then chosen for mass production.

For face shields, after receiving various demands from a myriad of Hospitals, we started with Hígia’s [12] and Prusa’s RC1 and RC2 models [13]. After users’ feedback and engineers’ analysis, we choose Prusa’s RC2 model with adaptations. Most items designed during the COVID-19 pandemic were tailored for the FFF process [4]. In our facilities, two selective laser sintering (SLS) machines were available for parts production. SLS is a powder bed fusion process that uses powder feedstock and has a major advantage to avoid support structures that allow stacking parts and increase productivity [14]. Therefore, since FFF and SLS are different processes with intrinsic characteristics, a new model design for productivity was developed. It is important to remark that, beyond the 3D printed face shield frames, the plastic sheets and elastic straps were provided by companies who gently donated these materials. The plastic sheets were laser cut as a service altruistically donated by a local company.

Regarding the *Charlotte* valves, we worked in partnership with the Brazilian Health Expeditionary NGO [15] and medics working directly with COVID-19 patients. Following ISINNOVA’s guidelines [7], we started replicating the original *Charlotte* valve design. Again, FFF was the suggested fabrication process because of its accessibility, and modifications in the design were necessary to improve the functionality of the part and build packaging, folding productivity in 1.6× compared with the original design. The new model presented no air leakage when coupled to Decathlon’s *Surface Snorkeling Mask Easybreath^®^* and is currently being used for noninvasive ventilation in COVID-19 patients in various Brazilian Hospitals [15]. More information regarding the valve production, optimization and tests can be found in one of our previously published papers [16].

## The future of 3D printing

The COVID-19 pandemic showed that the conventional production chain and distribution systems can be extremely fragile. In this regard, 3D printing proved as a powerful tool for decentralized, adaptive, flexible and versatile production with strong collaboration among countries. The trend of open-source technologies and design data sharing reached a new level as manufacturers, groups, universities and companies put common welfare over individual interests. For example, the ISINNOVA company patented its valves to let them be fabricated and used without legal issues [7]. From these actions and results, governments must create guidelines and special laws for emergencies to protect and encourage people to help.

As it is a relatively new technology, 3D printing did not reach its full potential. New materials in polymers, metals, ceramics and composites classes are being developed and opening new applications. For example, materials with bactericide properties [17] are currently being developed for 3D printing and will soon reach the general public. Furthermore, 4D printing, which uses smart materials as feedstock, will address several issues and leverage the number of solutions in medicine [18]. Bioprinting, a variant technology of 3D printing, promises to produce organs and tissues in the future and can currently create 3D *in vitro* models that help researchers understand disease spread in tissues and speed up the development of specific drugs [19]. Finally, the 3D printing particularities, such as layer-by-layer manufacturing, thermal cycle and residual stress, summed to its freedom of design, correlate to design for the additive manufacturing research area, which is strongly impacting the products, cost and productivity of the advanced manufacturing in all areas of industry [20].

## Final remarks

In this Editorial, we quickly highlight the role that 3D printing assumed during the COVID-19 pandemic worldwide and locally, specifically in our research lab. The advantages of the technique, such as freedom of design and rapid production of prototypes and even final parts, summed to its accessibility and open design, made it an essential feature for fighting off the pandemic. In this context, we strongly believe that its gained notoriety and all the current developments in the 3D printing field will make it a more established manufacturing technique in the industry and society and a powerful ally for future emergencies.
